# Endoscopic clipping of a complicated esophageal perforation following pneumatic dilation for achalasia: A case study and literature review

**DOI:** 10.1002/ccr3.6620

**Published:** 2022-11-27

**Authors:** Mohammad Minakari, Nahad Sedaghat

**Affiliations:** ^1^ Division of Gastroenterology, Department of Internal Medicine, School of Medicine Isfahan University of Medical Sciences Isfahan Iran; ^2^ School of Medicine Isfahan University of Medical Sciences Isfahan Iran

**Keywords:** achalasia, case report, endoscopic clipping, esophageal perforation, pneumatic balloon dilation

## Abstract

A transmural esophageal tear complicated by large size and unusual location was diagnosed in a 59‐year‐old man after undergoing pneumatic balloon dilation for achalasia. It was closed with six endo‐clips. The patient recovered and was discharged with ordinary diet 8 days later, after receiving supportive care for a week.

## Graphical abstract

Complicated esophageal perforations could also be managed with endoscopic intervention. As this depends on timely diagnosis, our key clinical message is that a careful endoscopic evaluation of the area and physical examination after PBD could obviate the need for later open surgery, reducing the morbidity associated with post‐PBD esophageal perforation.

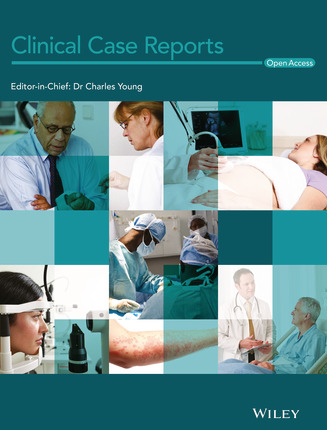

## 1 INTRODUCTION

In recent decades, pneumatic balloon dilation (PBD)—forceful relaxation of the lower esophageal sphincter (LES) muscles using dilatable balloons—has been a well‐established method for the treatment of achalasia. Being followed by a significant decrease in LES pressure, PBD has shown to be associated with a remarkable resolution of achalasia symptoms. In <5% of cases, however, PBD leads to esophageal perforation—a complication linked to severe morbidity.^1^ This complication could be managed with a wide variety of options, including conservative management^2^, ^3^ and surgical interventions.^4^, ^5^ Few recent reports point that post‐PBD esophageal tears could be managed with endoscopic clipping^6^, ^7^, ^8^, ^9^ or stenting^10^ as well, subject to timely diagnosis. Choosing the most beneficial treatment option for post‐PBD esophageal perforation is subject to careful assessment of the tear's size and shape, the effectiveness, the associated risk, and further sequelae of the chosen method. Non‐ or minimally‐invasive methods are currently preferred for uncomplicated cases; they are associated with non‐inferior effectiveness,^11^ faster recovery times, and less post‐interventional sequelae than open surgery.^12^ Surgical interventions are reserved for more severe cases with wider defects, pneumothorax or pneumomediastinum, serositis, deteriorating medical condition, and sepsis—as it has been the longest established method with lowest historical failure rates. Still, timely diagnosis before termination of the PBD procedure could enable the usage of endoscopic methods, preventing the mentioned sequalae and eventually obviating the need for open surgery.^11^ In the current study, we aimed to contribute to future evidence‐driven practice by reporting our own experience and providing an overview on the currently available options and their associated outcomes.

## 2 CASE STUDY

In April 2018, a 59‐year‐old man presented to the gastroenterology clinic of the Alzahra University Hospital complaining of a progressive dysphagia to liquid and solid diet. The onset of his symptoms dated back to 2 years before presentation, when he started to experience a gradually worsening sensation of chest pain and retrosternal fullness when consuming solid food. Ever since, he mentioned that he had to eat very slowly and consume a lot of fluids with his meals. As his symptoms progressed, he experienced the same sensations while consuming liquid diet as well.

**FIGURE 1:**
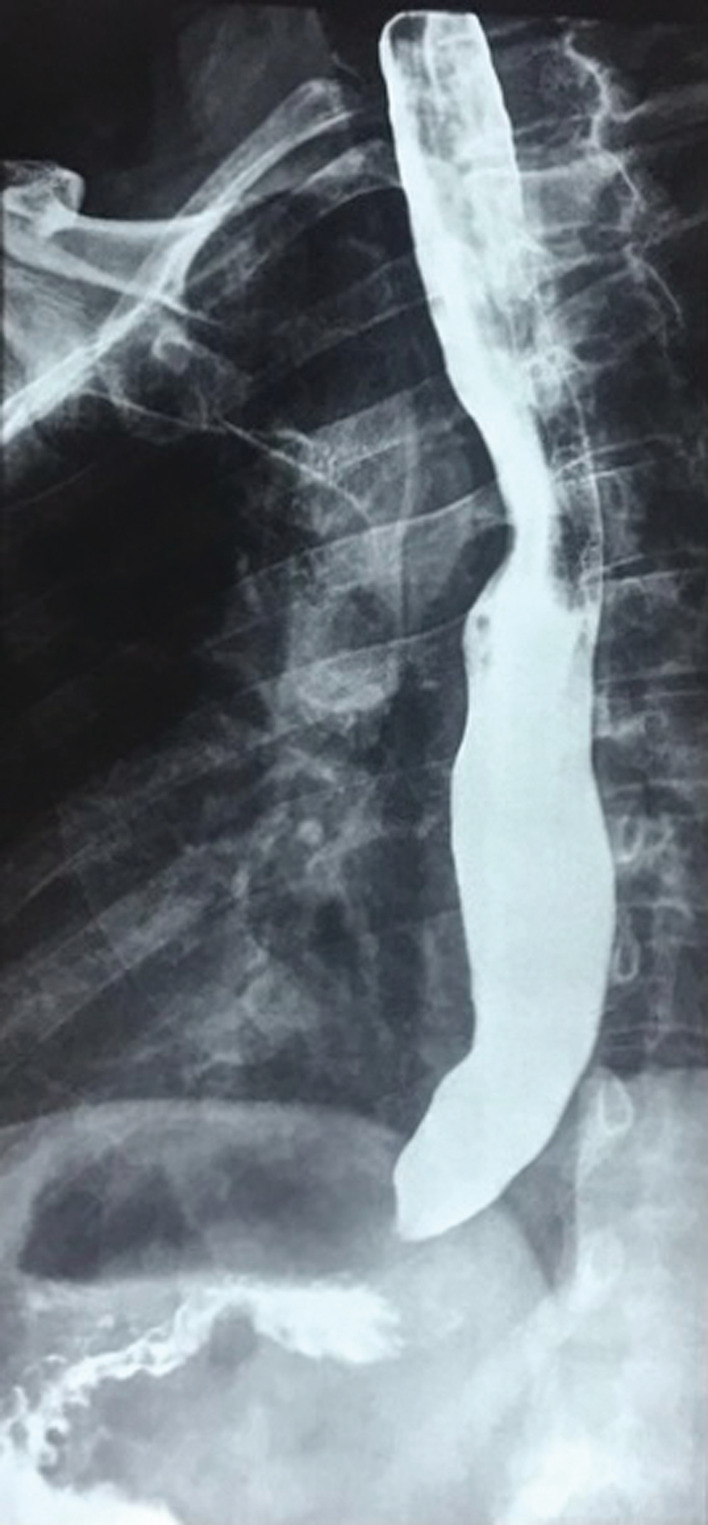
Upper gastrointestinal radiograph after barium swallow showing a typical “bird‐beak” view indicative of achalasia

Upon presentation, physical examination and laboratory tests were suggestive of a mild anemic state and otherwise unremarkable. Initial endoscopic evaluation revealed evidence of esophageal candidiasis along with a severe constriction in the LES region. His esophageal candidiasis resolved 7 days later after treatment with oral fluconazole (100 mg, BID). He was eventually diagnosed with achalasia based on typical findings in his history of illness, follow‐up upper gastrointestinal endoscopy, and upper gastrointestinal X‐ray series (Figure 1). Although determining the manometric subtype of achalasia using high‐resolution manometry has some relevance in practice, mainly for finding the type‐III cases who would benefit the most from per‐oral endoscopic myotomy (POEM),^13^ high‐resolution manometry was not performed for the patient as POEM was practically unavailable in the region. After careful assessment of the situation, the patient was offered with PBD and surgical myotomy while being provided with a detailed description of the pros and cons of each; he later chose to undergo PBD. He was therefore, admitted and prescribed with an only‐clear‐liquid preparation diet, before undergoing PBD 2 days later.

**FIGURE 2:**
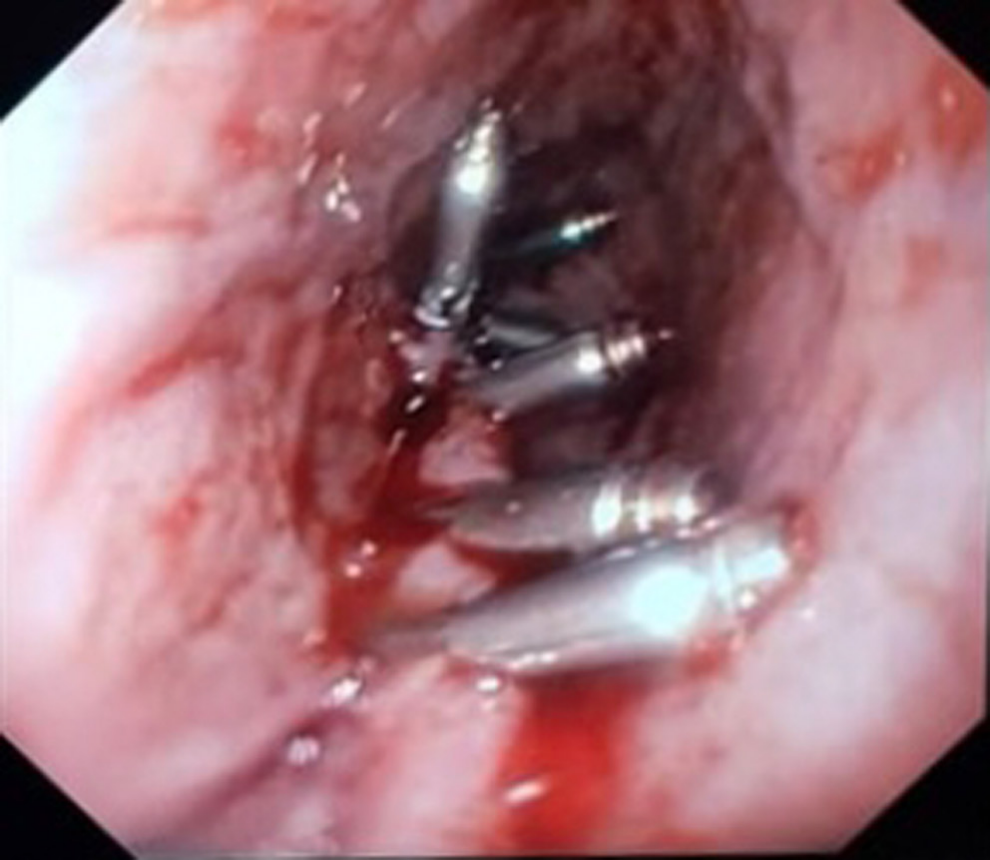
Direct endoscopic view of the patient's post‐PBD transmural esophageal tear closed with six endo‐clips

The PBD procedure was initiated by sedating the patient with midazolam (15 mcg/kg, IV) and propofol (2 mg/kg, IV). His vital signs were being monitored throughout the procedure by the team of anesthesiologists and gastroenterologists who were performing it. Under direct endoscopic vision, the scope was passed to the stomach and second portion of duodenum, facilitating the placement of a guide wire in the gastric antrum. A 30 mm achalasia balloon dilator (Boston Scientific) was thereafter, passed into the esophagus on the guide wire. After being placed in the proper site under direct endoscopic vision, the balloon dilator was inflated for 60 s with 10 PSI pressure per protocol. It was then successfully deflated and withdrawn. Immediate endoscopic inspection after dilation revealed what appeared to be a 3 cm linear, deep mucosal tearing at the left lateral side of the lower esophagus. Closer inspection revealed that it was a complete transmural tear of the esophageal wall. Transmural esophageal perforation was immediately diagnosed and confirmed, after the patient developed subcutaneous emphysema at the anterolateral sides of his chest and neck. Although large, the tear was linear and seemed amenable to endoscopic repair; hence, the team decided to attempt for an endoscopic closure of the tear using endo‐clips. First, the distal margin of the tear was closed to prevent any further widening, and then, it was closed in an upward direction. A total of six clips were used to close the entire tear (Figure 2). After that, a nasogastric tube was placed for the patient under endoscopic guide, and the procedure was terminated.

Meanwhile, the patient started to show symptoms of respiratory failure; therefore, he was intubated after the procedure, was transferred to intensive care unit (ICU), and was prescribed with intravenous (IV) fluids and antibiotics. There, he had subcutaneous emphysema in his chest, neck, and face. A subsequent chest radiograph 4 h after the procedure revealed left pneumothorax, which was managed with a chest tube placed in the fifth intercostal space. He was then put on mechanical ventilation while being closely observed in the ICU. 24 h later, he regained spontaneous ventilation. He was therefore, extubated, and did not show any sign of respiratory distress thereafter. After 6 days of parenteral diet upper gastrointestinal X‐ray series and computer tomography studies were performed, revealing no leakage. Hence, oral liquid diet was initiated, which was well tolerated. On day seven post‐PBD, regular diet was resumed, which was well tolerated as well without any complications. On day eight post‐PBD, after a follow‐up chest radiograph confirmed complete expansion of the left lung, the chest tube was removed and replaced with a petroleum sterile gauze dressing, and the patient was discharged. In a follow‐up visit in July 2018 (3 months post‐PBD), the patient reported complete resolution of his achalasia symptoms, without any further sequelae.

## 3 DISCUSSION AND LITERATURE REVIEW

We reported a case of a PBD‐associated transmural esophageal tear with a large size and in an unusual location causing pneumothorax and subcutaneous emphysema. The tear was managed with endoscopic clipping and supportive care and the patient recovered without sequalae in a week. This case could be regarded as a replicative highlight of the effectiveness of endoscopic intervention for management of esophageal perforation^6^, ^7^, ^8^, ^9^—even for cases complicated by unusual tear location and size, subsequent pneumothorax, and subcutaneous emphysema. Still, the effectiveness and safety of this method is, dependent on immediate diagnosis—before factors such as superinfections or serositis further complicate the case. An exhaustive post‐dilation endoscopic inspection of the area along with a physical examination focusing on possible subcutaneous emphysemas could, therefore, obviate the need for later open surgery in cases of PBD‐associated esophageal perforation.

Furthermore, we reviewed all of the reported cases of post‐PBD transmural esophageal perforations in MEDLINE and Google Scholar using the keywords “Pneumatic balloon dilation,” “achalasia,” “transmural tear,” and “esophageal perforation”—with proper Boolean operators and specific vocabulary considerations. Conservative management of esophageal perforation with IV fluids and antibiotics has been effective with minimal recovery times in contained perforations.^14^, ^15^ Even for complicated cases with pleural/pericardial effusions, conservative management plus pleural/pericardial drainage tubes may be adequate^2^—although the mentioned cases are suggested to undergo prompt surgery.^3^ Furthermore, a randomized porcine study^16^ on experimental esophageal perforation models indicated similar outcomes with surgical and endoscopic interventions, yet endoscopic methods took significantly less time. In humans, open surgical intervention has been associated with the least failure rate among all of the reported cases.^5^, ^12^, ^17^, ^18^ Laparoscopic intervention, although used to show a relatively high failure rate in earlier studies,^5^ has been associated with high success rate in more recent studies.^4^, ^12^ Endoscopic methods such as stenting, clipping, and suturing have also been effective and safe in uncomplicated cases with small perforations mostly located posteriorly^6^, ^8^, ^9^, ^10^, ^19^, ^20^, ^21^—although failure has been reported in earlier cases.^21^ In addition to mild uncomplicated cases, our experience shows that endoscopic repair could be an option for cases of large lateral esophageal perforations complicated with pneumothorax as well. Nevertheless, the current evidence among humans is limited to case studies; randomized human studies are warranted to enable proper evidence‐driven management of PBD‐associated esophageal perforation. Additionally, in all cases regardless of the chosen management method, close observation and clinical monitoring have proven crucial for timely identification of deteriorating states and prevention of harm; therefore, clinicians are encouraged to closely observe and monitor their patients regardless of the treatment they receive—especially until higher‐level evidence becomes available.

## 4 CONCLUSION

We reported our experience in managing a complicated PBD‐associated esophageal perforation case with endoscopic clipping and supportive care. Compared with other PBD‐associated esophageal perforation cases reported in the literature which were managed with endoscopic interventions, our case was unique in terms of its associated complications, that is, the location and size of the tear, and subsequent development of pneumothorax and subcutaneous emphysema. In addition to surgery—and subject to confirmation by stronger studies—endoscopic intervention could be regarded as a safe and effective option for the uncomplicated PBD‐associated esophageal perforations, as well as the cases with the mentioned complications. As the usage of this method depends on prompt diagnosis, the key clinical message in this case is that an exhaustive endoscopic inspection of the area along with physical examination after PBD could obviate the need for later open surgery, reducing the morbidity associated with post‐PBD esophageal perforation.

## AUTHOR CONTRIBUTIONS

MM has managed the case and gathered the relevant case information, validated the findings, and drafted the initial draft. NS has searched databases for relevant data, reviewed the relevant literature, and reviewed and edited the manuscript. Both of the authors have reviewed the final manuscript and approved it for publication.

## ETHICAL APPROVAL

In accordance with national guidelines, a written informed consent was obtained from the reported patient for publishing his anonymized case along with any unidentifiable images.

## CONSENT

Written informed consent was obtained from the patient to publish this report in accordance with the journal's patient consent policy.

## Data Availability

No data was produced in this study additional to what is presented.

## References

[1] Felix VN . Results of pneumatic dilation in treating achalasia: predictive factors. Ann N Y Acad Sci. 2018;1434(1):124‐131. doi:10.1111/nyas.13844 29766515

[2] Vanuytsel T , Lerut T , Coosemans W , et al. Conservative management of esophageal perforations during pneumatic dilation for idiopathic esophageal achalasia. Clin Gastroenterol Hepatol. 2012;10(2):142‐149. 22064041 10.1016/j.cgh.2011.10.032

[3] Madanick RD . Medical management of iatrogenic esophageal perforations. Curr Treat Options Gastroenterol. 2008;11(1):54‐63. 21063864 10.1007/s11938-008-0007-9

[4] Sánchez‐Pernaute A , Aguirre EP , Talavera P , et al. Laparoscopic approach to esophageal perforation secondary to pneumatic dilation for achalasia. Surg Endosc. 2009;23(5):1106‐1109. 18814004 10.1007/s00464-008-0114-7

[5] Hunt DR , Wills VL , Weiss B , Jorgensen JO , DeCarle DJ , Cook IJ . Management of esophageal perforation after pneumatic dilation for achalasia. J Gastrointest Surg. 2000;4(4):411‐415. 11058860 10.1016/s1091-255x(00)80021-9

[6] Coda S , Antonellis F , Tsagkaropulos S , Francioni F , Trentino P . Complete endoscopic closure (clipping) of a large esophageal perforation after pneumatic dilation in a patient with achalasia. J Laparoendosc Adv Surg Tech A. 2012;22(8):815‐818. doi:10.1089/lap.2012.0198 22973857

[7] Lázár G , Paszt A , Mán E . Role of endoscopic clipping in the treatment of oesophageal perforations. World J Gastrointest Endosc. 2016;8(1):13‐22. 26788259 10.4253/wjge.v8.i1.13 PMC4707319

[8] Schneider AR , Schepp W . Sp951 endoscopic closure of acute esophageal perforation following pneumatic dilation for achalasia. Gastrointest Endosc. 2012;75(4):AB110.

[9] Chaudhary S , Azizi S , Nietsch H , Sood V . Case series of successful endoscopic closures of iatrogenic perforations using the over‐the‐scope‐clip (OTSC): 1532. Am J Gastroenterol. 2013;108:S458.

[10] Elhanafi S , Othman M , Sunny J , et al. Esophageal perforation post pneumatic dilatation for achalasia managed by esophageal stenting. Am J Case Rep. 2013;14:532‐535. doi:10.12659/ajcr.889637 24349606 PMC3862140

[11] Ghoshal UC , Karyampudi A , Verma A , et al. Perforation following pneumatic dilation of achalasia cardia in a university hospital in northern India: a two‐decade experience. Indian J Gastroenterol. 2018;37(4):347‐352. doi:10.1007/s12664-018-0874-5 30121890

[12] Fry LC , Mönkemüller K , Neumann H , Schulz HU , Malfertheiner P . Incidence, clinical management and outcomes of esophageal perforations after endoscopic dilatation. Z Gastroenterol. 2007;45(11):1180‐1184. doi:10.1055/s-2007-963558 18027320

[13] Khashab MA , Vela MF , Thosani N , et al. ASGE guideline on the management of achalasia. Gastrointest Endosc. 2020;91(2):213‐27.e6. doi:10.1016/j.gie.2019.04.231 31839408

[14] Swedlund A , Traube M , Siskind BN , McCallum RW . Nonsurgical management of esophageal perforation from pneumatic dilatation in achalasia. Dig Dis Sci. 1989;34(3):379‐384. 2920644 10.1007/BF01536259

[15] Gershman G , Ament ME , Vargas J . Frequency and medical management of esophageal perforation after pneumatic dilatation in achalasia. J Pediatr Gastroenterol Nutr. 1997;25(5):548‐553. 9360212 10.1097/00005176-199711000-00012

[16] Fritscher‐Ravens A , Hampe J , Grange P , et al. Clip closure versus endoscopic suturing versus thoracoscopic repair of an iatrogenic esophageal perforation: a randomized, comparative, long‐term survival study in a porcine model (with videos). Gastrointest Endosc. 2010;72(5):1020‐1026. doi:10.1016/j.gie.2010.07.029 21034902

[17] Urbani M , Mathisen DJ . Repair of esophageal perforation after treatment for achalasia. Ann Thorac Surg. 2000;69(5):1609‐1611. 10881864 10.1016/s0003-4975(00)01149-8

[18] Kim H . Gastroplasty for esophageal perforation after endoscopic balloon dilatation for achalasia: two cases. J Korean Med Sci. 2014;29(5):739‐742. doi:10.3346/jkms.2014.29.5.739 24851034 PMC4024952

[19] Oude Nijhuis RA , Bergman JJ , Takkenberg RB , Fockens P , Bredenoord AJ . Non‐surgical treatment of esophageal perforation after pneumatic dilation for achalasia: a case series. Scand J Gastroenterol. 2020;55(10):1248‐1252. 32924655 10.1080/00365521.2020.1817541

[20] Fischer A , Schrag H , Goos M , Von Dobschuetz E , Hopt U . Nonoperative treatment of four esophageal perforations with hemostatic clips. Dis Esophagus. 2007;20(5):444‐448. 17760660 10.1111/j.1442-2050.2007.00652.x

[21] Siersema PD , Homs MY , Haringsma J , Tilanus HW , Kuipers EJ . Use of large‐diameter metallic stents to seal traumatic nonmalignant perforations of the esophagus. Gastrointest Endosc. 2003;58(3):356‐361. 14528208 10.1067/s0016-5107(03)00008-7

